# Physiological and Pathophysiological Relevance of the Anion Transporter Slc26a9 in Multiple Organs

**DOI:** 10.3389/fphys.2018.01197

**Published:** 2018-08-28

**Authors:** Xuemei Liu, Taolang Li, Biguang Tuo

**Affiliations:** ^1^Department of Gastroenterology, Affiliated Hospital, Zunyi Medical University, Zunyi, China; ^2^Digestive Disease Institute of Guizhou Province, Zunyi, China; ^3^Department of Thyroid and Breast Surgery, Affiliated Hospital, Zunyi Medical University, Zunyi, China

**Keywords:** Slc26a9, multiple organs, expression pattern, physiological function, pathophysiology, health and disease

## Abstract

Transepithelial Cl^-^ and HCO_3_^-^ transport is crucial for the function of all epithelia, and HCO_3_^-^ is a biological buffer that maintains acid-base homeostasis. In most epithelia, a series of Cl^-^/HCO_3_^-^ exchangers and Cl^-^ channels that mediate Cl^-^ absorption and HCO_3_^-^ secretion have been detected in the luminal and basolateral membranes. Slc26a9 belongs to the solute carrier 26 (Slc26) family of anion transporters expressed in the epithelia of multiple organs. This review summarizes the expression pattern and functional diversity of Slc26a9 in different systems based on all investigations performed thus far. Furthermore, the physical and functional interactions between Slc26a9 and cystic fibrosis transmembrane conductance regulator (CFTR) are discussed due to their overlapping expression pattern in multiple organs. Finally, we focus on the relationship between *slc26a9* mutations and disease onset. An understanding of the physiological and pathophysiological relevance of Slc26a9 in multiple organs offers new possibilities for disease therapy.

## Introduction

Acid/base homeostasis is vital for life because protein and enzyme functions, cell structures and membrane permeability are altered by changing pH. In most epithelia, transepithelial chloride absorption and bicarbonate secretion are associated with fluid secretion, which is important for normal functioning. Slc26 anion exchangers are members of a recently discovered gene family with multifunctional transmembrane proteins. These proteins mediate the transport of various monovalent and divalent anions, including chloride, bicarbonate, sulfate, iodide and oxalate, mainly across the plasma membrane of epithelial cells and contribute to the composition and pH of secreted fluids in the body (El Khouri and Toure, 2014). This gene family consists of 11 members and is divided into three groups, e.g., selective sulfate transporters, Cl^-^/HCO_3_^-^ exchangers and anion channels, based on their proven or putative transport properties (Mount and Romero, 2004; Dorwart et al., 2008; Ohana et al., 2009; Alper and Sharma, 2013). Slc26a9 is classified into the third group and expressed in the apical membrane of multiple organs to regulate Cl^-^ and HCO_3_^-^ transport by different ion transport methods. In this review, we discuss the current knowledge of the expression pattern, functional diversity, regulatory mechanism and pathophysiology of this novel protein in different organs.

### Cloning, Expression and Physiological Functions of Slc26a9 in Different Organs

Slc26a9 (solute carrier family 26 member 9) is a member of the Slc26 family of multifunctional anion transporters. Slc26a9 was cloned on the basis of its homology to other Slc26 isoforms. In humans, SLC26A9 maps to chromosome 1 and encodes a 791-amino-acid protein (Lohi et al., 2002). SLC26A9 (human)/Slc26a9 (mouse) is expressed at high levels in the lung (Lohi et al., 2002) and stomach (Xu et al., 2008; Liu et al., 2015), to some extent in the proximal duodenum, and at low levels in the distal duodenum and pancreas (Liu et al., 2015). Slc26a9 is also detected in some specialized cells in the kidney (Amlal et al., 2013), neural system (Chang et al., 2009b), reproductive tract (Dorwart et al., 2007), salivary gland (Tandon et al., 2017), and prostate (Lohi et al., 2002). Although Slc26a9 is not expressed in normal enamel cells (Lacruz et al., 2012), it is upregulated in Slc26a1 and Slc26a7 null mice, suggesting that it plays a strong compensatory role during enamel maturation (Yin et al., 2015). Functional studies showed that Slc26a9 is involved in diverse transport modes in different systems. First, recent work in *Xenopus* oocytes, Fischer rat thyroid epithelial cells, HEK cells and COS-7 cells expressing Slc26a9 has shown that Slc26a9 is a highly selective Cl^-^ channel with minimal OH/HCO_3_ permeability that is regulated by with-no-lysine kinases (WNK) and cystic fibrosis transmembrane conductance regulator (CFTR) in heterologous cells (Dorwart et al., 2007; Loriol et al., 2008; Bertrand et al., 2009; Salomon et al., 2016). Loriol et al. reported that the conductivity of Slc26a9 is enhanced at high HCO_3_^-^ concentrations (Loriol et al., 2008). Second, electrophysiological experiments involving *Xenopus* oocytes, HEK cells, and animal models have attributed the following three different transport modes to Slc26a9: Cl^-^ channel (Loriol et al., 2008; Bertrand et al., 2009; Avella et al., 2011; Ousingsawat et al., 2012; Amlal et al., 2013), Cl^-^/HCO_3_^-^ exchanger (Xu et al., 2005; Demitrack et al., 2010) and Na^+^ transporter (Chang et al., 2009b). Additionally, the Slc26a9 protein plays multiple physiological roles in the transport of several anions, including I^-^, NO3, gluconate, SO_4_^2-^, and Br^-^, at different conductances (Dorwart et al., 2007; Loriol et al., 2008).

### Slc26a9 Functions as a Cl^-^ Channel in the Airway

Slc26a9 is predominantly expressed in the apical membrane of alveolar, tracheal, and bronchiolar epithelial cells in the lung (Lohi et al., 2002; Bertrand et al., 2009; Liu et al., 2014). Initial studies have shown that Slc26a9 contributes to constitutive and cyclic adenosine monophosphate (cAMP)-dependent Cl^-^ secretion in cultured human bronchial epithelial (HBE) cells, and Slc26a9 has been suggested to functionally interact with CFTR *in vitro* (Bertrand et al., 2009; Avella et al., 2011), indicating that Slc26a9 may serve as a chloride channel that compensates for CFTR dysfunction in CF. Based on the functional properties of Slc26a9 in transduced cells and its expression pattern in human and mouse airways (Lohi et al., 2002; Chang et al., 2009b), questions regarding the function of Slc26a9 in maintaining airway surface liquid (ASL) homeostasis in health and allergic airway disease have emerged. Studies investigating the function of Slc26a9 in native epithelia have been performed in the lung of Slc26a9 null mice, and the essential role of Slc26a9 in mediating Cl^-^ secretion and preventing mucus obstruction during airway inflammation induced by IL-13 instillation (Anagnostopoulou et al., 2012), but not under physiological conditions, has been identified. This finding suggests that anion conductance, which was found to be upregulated in allergic airway inflammation in a previous study (Anagnostopoulou et al., 2010), may indeed occur through Slc26a9 and that the inability to activate this conductance may result in an increase in airway pathology. Taken together, these data indicate that besides TMEM16A and CFTR, Slc26a9 functions as an alternative chloride channel that may contribute to the regulation of ASL, which is essential for mucus clearance under pathophysiological conditions in the airways.

### Slc26a9 Is Involved in Various Ion Transport Systems in the Digestive System

#### In the Stomach

Slc26a9 is highly expressed in the apical membrane of parietal cells, surface epithelial cells and deep cells in the gastric glands and is co-localized with H^+^/K^+^-ATPase expression (Xu et al., 2005, 2008). The initial characterization of Slc26a9 transport by the measurements of intracellular pH (pH_i_) suggested that Slc26a9 can function as a Cl/HCO_3_ exchanger in gastric surface cells to support HCO_3_^-^ secretion in the stomach, which can be inhibited by NH4^+^ *in vivo* (Xu et al., 2005). These results raise the question of whether the expression of Slc26a9 is reduced during *Helicobacter pylori* (*H. pylori*, Hp) infection due to the production of ammonia (NH3)/NH_4_^+^ through its urease activity, which may predispose patients to acidic injury and peptic ulcers by impairing Slc26a9-mediated gastric HCO_3_^-^ secretion. However, a subsequent study showed that the mRNA and protein expression levels of Slc26a9 are not altered in gastric surface epithelial cells in Hp-infected mice, which might be a compensatory response to overcome the chronic inhibition of Slc26a9-mediated HCO_3_^-^ secretion by the Hp infection (Henriksnas et al., 2006). Subsequently, Demitrack et al. demonstrated that Slc26a9 can activate cellular HCO_3_^-^ secretion through its anion transporter if the gastric epithelium is damaged (Demitrack et al., 2010). Collectively, these observations suggest that Slc26a9 functions as a Cl^-^/HCO_3_^-^ exchanger. However, based on a Slc26a9-deficient mouse model, functional experiments have shown that the deletion of Slc26a9 results in impaired acid secretion, indicating that Slc26a9 can function as a Cl^-^ channel (Xu et al., 2008). Moreover, the absence of Slc26a9 expression in the murine stomach at young age causes parietal cell loss, hypochlorhydria and massive fundic hyperplasia (Xu et al., 2008), suggesting that the Slc26a9 gene is essential for parietal cell function and survival. Loss of parietal cells is known to contribute to a premalignant environment in the gastric mucosa, and gastric cancer normally develops in the setting of parietal cell loss and mucous cell metaplasia (Goldenring and Nam, 2010). Thus, Slc26a9 deficiency may promote gastric carcinogenesis and should be intensively investigated.

#### In the Duodenum

Recently, polymorphisms in the *slc26a9* gene were associated with an increased risk of meconium ileus (MI) in infants with cystic fibrosis (CF) (Sun et al., 2012), which addresses the question regarding the expression and function of Slc26a9 in the intestine. Early expression data showed that low levels of Slc26a9 were detected in the duodenum (Xu et al., 2005). However, a significant reduction in basal duodenal HCO_3_^-^ secretion was observed in Slc26a9-deficient duodenum *in vivo*, and forskolin-induced stimulation did not alter HCO_3_^-^ secretion. Additionally, both luminal low acid and prostaglandin E2 (PGE2) treatments strongly reduced HCO_3_^-^ secretion in the absence of Slc26a9 expression, suggesting that Slc26a9 plays an important role in orchestrating the acid-induced duodenal HCO_3_^-^ secretory response (Singh et al., 2013). Subsequently, a more precise study found that Slc26a9 was detected in the proximal duodenum but not in the more distal part of the intestine where MI occurs (Liu et al., 2015). The loss of Slc26a9 expression resulted in reduced survival of CFTR-deficient mice and strongly impaired HCO_3_^-^ and fluid secretion in the proximal duodenum, particularly at a young age, indicating that Slc26a9 plays a key role in regulating HCO_3_^-^ secretory and fluid absorptive changes in the proximal duodenal mucosa despite the low Slc26a9 expression at the whole organ level (Liu et al., 2015). The association between Slc26a9 and MI might be related to the absence or malfunction of Slc26a9 in the upper GI tract, which results in maldigestion and impaired downstream signaling. More importantly, we depict the following four potential ion transport modes of Slc26a9 in the duodenum: a. Slc26a9 is located in the crypts of the duodenum and functions as a chloride channel (similar to CFTR) to facilitate Cl^-^ recycling via another Cl^-^/HCO_3_^-^ exchanger, b. Slc26a9 functions as an anion exchanger that is functionally coupled to CFTR to regulate HCO_3_^-^ secretion, c. Slc26a9 and CFTR interact structurally and/or functionally in a positive fashion to enhance HCO_3_^-^ secretion, and d. Slc26a9 is a crypt enterocyte anion channel that functions indirectly in transepithelial anion transport but is possibly involved in volume control/apoptosis/migration/differentiation. In this case, the altered ion transport could be a secondary phenomenon based on the changes in cellular growth and differentiation patterns. Therefore, the importance of Slc26a9 in the regulation of HCO_3_^-^ secretion may offer a new possibility for the protection of duodenum ulcer, as HCO_3_^-^ secretion is essential for maintaining acid/base homeostasis and mucosal protection in the duodenum.

#### In the Pancreas and Biliary System

Polymorphisms in Slc26a9 are associated with an increased incidence and poor prognosis of diabetic individuals with CF (Blackman et al., 2013), However, knowledge of the expression and function of Slc26a9 in the pancreas and biliary system is lacking. We found that Slc26a9 was expressed in the pancreatic parenchyma, liver, gallbladder and microdissected pancreatic and bile ducts at low levels (Liu et al., 2015). Afterward, functional data showed that the deletion of Slc26a9 is associated with a reduction in pancreatic fluid, but not HCO_3_^-^ secretion, in young female mice. This finding demonstrates that Slc26a9 plays a role in pancreatic ductal physiology despite the fairly low expression levels compared to those of CFTR and other electrolyte transporters. Furthermore, a slight reduction in the normalization of blood glucose levels was observed after an intravenous glucose bolus in older female mice. The female preponderance for an Slc26a9-related loss of ductal function is interesting in the context of the higher incidence of diabetes in female CF patients (Li et al., 2016). Thus, Slc26a9 can function as an alternative chloride channel to regulate fluid absorption and glucose metabolism in the pancreas, suggesting that Slc26a9 may not only be involved in CF-related diabetes (CFRD) onset but also may contribute to the occurrence of Type 2 diabetes.

#### Role of Slc26a9 in the Kidney

**FIGURE 1:**
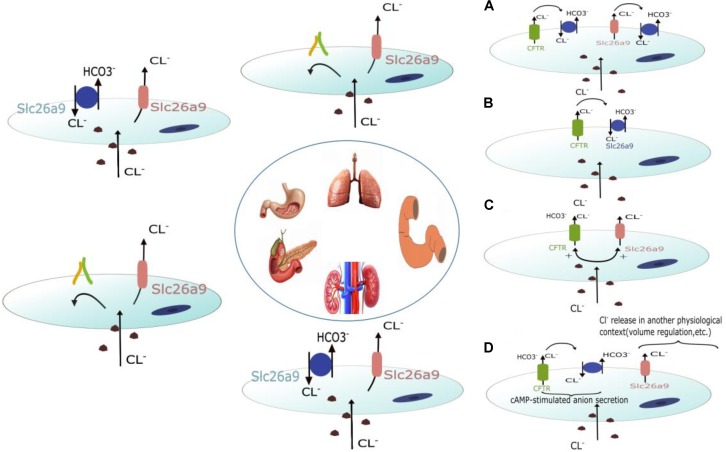
Schematic diagram depicting the potential roles of Slc26a9 in different organs. Slc26a9 functions as a Cl^-^ channel in the lung and pancreases. In the stomach and kidney, Slc26a9 can function as both a Cl^-^ channel and Cl^-^/HCO_3_^-^ exchanger. In addition to functioning as a Cl^-^ channel and Cl^-^/HCO_3_^-^ exchanger, four potential roles of Slc26a9 in the duodenum have been discussed in other organs. Slc26a9 and CFTR may interact structurally and/or functionally in a positive fashion to enhance HCO_3_^-^ secretion. Slc26a9 is a crypt enterocyte anion channel that functions indirectly with regard to transepithelial anion transport and is possibly involved in volume control/apoptosis/migration/differentiation.

Slc26a9 is mainly found in the medulla and cultured medullary collecting duct cells and is located on the apical membrane of a subset of cells in the outer medullary collecting duct (OMCD), the initial portion of the inner medullary collecting duct (IMCD), and principal cells (Amlal et al., 2013; Soleimani, 2013). Functional analyses showed that the absence of Slc26a9 in mouse results in impaired chloride and sodium excretion after exposure to a high-salt diet or water deprivation. This impairment is accompanied by elevated systemic arterial pressure, suggesting that Slc26a9 functions as a Cl^-^ channel that regulates renal salt excretion and blood pressure (Amlal et al., 2013). However, RT-PCR analyses and pHi measurements in cultured proximal tubular cell lines (PTE) from Wistar Kyoto (WKY) and spontaneously hypertensive rats (SHR) showed that Slc26a9 is upregulated in SHR PTE cells. Slc26a9 functions as a Cl^-^/HCO_3_^-^ exchanger that mediates part of Cl^-^ and HCO_3_^-^ transport activity in both WKY and SHR cells (Simao et al., 2013). Therefore, the exact ion transport mode of Slc26a9 in the kidney is still controversial. **Figure 1** depicts the potential ion transport modes of Slc26a9 in different organs.

### Reciprocal Interaction Between Slc26a9 and CFTR

Several Slc26a9 regulatory mechanisms, including transcription, protein trafficking, post-translational modifications, macro-molecular complex formation (Dorwart et al., 2008) and WNK regulation (Dorwart et al., 2007; Park et al., 2012; Bazúa-Valenti et al., 2015), have been described. The reciprocal regulation between CFTR and Slc26a9 has also been extensively discussed (Dorwart et al., 2008; Fong, 2012; Li et al., 2017) due to the overlapping expression pattern and function of the two proteins in some organs. First, co-immunoprecipitation experiments conducted in HEK293 cells transiently expressing Slc26a9 and CFTR proteins demonstrated the existence of the Slc26a9-CFTR complex (Bertrand et al., 2009). This complex was also confirmed by co-immunoprecipitation in a CF bronchial epithelial cell line (CFBE4Io) stably expressing wild-type or p.phe508del CFTR proteins transduced with Slc26a9 (Avella et al., 2011). The direct interaction between the two proteins was further demonstrated by co-immunoprecipitation experiments using the purified Slc26a9 anti-sigma factor antagonist (STAS) domain and purified CFTR R domain (Chang et al., 2009a). Second, Slc26a9 interacts functionally and/or structurally with CFTR when co-expressed heterologously, but whether this interaction results in enhancement or inhibition is controversial. Some findings suggest that Slc26a9 can enhance CFTR-mediated anion secretion in cultured airway cells and *Xenopus* oocytes (Bertrand et al., 2009; Avella et al., 2011; Ousingsawat et al., 2012), and the reciprocal stimulation of both Slc26a9 and the CFTR current by an STAS-R domain interaction in a protein kinase A (PKA)-dependent fashion has been described. This finding is similar to previous descriptions of the interaction between the anion exchangers Slc26a3 and Slc26a6 (Bertrand et al., 2009). Furthermore, we confirmed that a deletion of Slc26a9 reduces survival in CFTR-deficient mice (Liu et al., 2015). Interestingly, Avella et al. also reported that the level of Slc26a9 protein increased in response to co-expression with the CFTR channel (Avella et al., 2011). In contrast, Slc26a9 has been found to inhibit (and be inhibited by) CFTR in non-polarized HEK293 cells (Ousingsawat et al., 2012). Additionally, a recent study showed that Slc26a9 is co-expressed with F508del CFTR, and its trafficking defect led to a PDZ motif-sensitive intracellular retention of Slc26a9 (Bertrand et al., 2017). Therefore, Slc26a9 function and regulation may be highly context-dependent and merit further investigation.

### SLC26A9 and Human Diseases

Recent reports have demonstrated that mutations in SLC26A proteins cause a variety of human diseases (Dorwart et al., 2008; El Khouri and Toure, 2014). In humans, pathogenic mutations in *slc26a9* have been associated with some diseases as well. Initially, based on a public single nucleotide polymorphism (SNP) database, Romero’s group found that *slc26a9* polymorphisms cause several functional modifications, including increased or decreased Cl^-^ current and Cl^-^/HCO_3_^-^ exchanger activity and altered protein expression, which could lead to a spectrum of pathophysiologies. However, SLC26A9 is not directly associated with any disease (Chen et al., 2012). Nevertheless, a genome-wide pharmacogenomics study investigating neurocognition reported that rs11240594 at *slc26a9* mediates the effects of olanzapine on processing speed, indicating that *slc26a9* may be a novel candidate gene for antipsychotic responses in schizophrenia (McClay et al., 2011). This study represents the only report associated with the neural system thus far. In the airways, an analysis of a database of a large European cohort of childhood asthma was performed, resulting in the detection of polymorphisms that carried an increased risk of childhood asthma. Indeed, a polymorphism (at rs2282430) in the *slc26a9* gene was found to be associated with an increased asthma incidence. Additionally, polymorphisms in the 3′ UTR of *slc26a9* that reduce protein expression *in vitro* are associated with asthma. These data provide initial evidence that SLC26A9 may be involved in asthma pathogenesis in humans, suggesting that SLC26A9 may serve as a therapeutic target for allergic airway diseases (Anagnostopoulou et al., 2012; Mall and Galietta, 2015; Sala-Rabanal et al., 2015). Subsequently, a study involving a cohort of 147 patients presenting with diffuse idiopathic bronchiectasis, corresponding to a common lung disease primarily induced in CF patients, was performed. The authors showed that two missense variants (p. Arg575Trp and p.Val486Ile) in the *slc26a9* gene can decrease Cl^-^ channel transport and are related to diffuse idiopathic bronchiectasis, indicating that SLC26A9 is a candidate gene for CF-like disease (Bakouh et al., 2013). Recently, more studies have shown that SNPs in the *slc26a9* gene are associated with CF-related disease onset, suggesting that SLC26A9 is a novel CFTR regulator. Sun and colleagues were the first to report that SNPs (at rs4077468) in the *slc26a9* gene were significantly associated with increases in MI in CF infants (Sun et al., 2012). Interestingly, another SNP (rs7512462) in the *slc26a9* gene was pleiotropic for MI, pancreatic insufficiency and early exocrine pancreatic disease in CF individuals (Li et al., 2014; Soave et al., 2014; Miller et al., 2015; Pereira et al., 2018) but not progressive lung disease patients (Wright et al., 2011; Li et al., 2014; Corvol et al., 2015). However, new data demonstrated that the rs7512462^∗^C allele is associated with a better response to ivacaftor (VX-770, C24H28N2O3) in patients with CF and mutations, resulting in improvements in pulmonary function (Pereira et al., 2017). This finding confirmed that Slc26a9 airway modification requires CFTR at the cell surface and that a common variant in Slc26a9 may predict the response to CFTR-directed therapeutics (Strug et al., 2016). Furthermore, Blackman’s group reported that two SNPs in complete linkage disequilibrium (rs4077468 and rs4077469) located within the slc26a9 gene were found to increase the risk of CFRD onset, but the same genetic alterations in *slc26a9* played a protective role against Type 2 diabetes predisposition (Blackman et al., 2013; Meyre and Pare, 2013). This finding suggests that the ductal fluid flow in the exocrine pancreas was changed due to abnormal chloride channel function resulting from CFTR mutations and that SNPs at the *slc26a9* locus play a substantial role in CFRD, further supporting that Slc26a9 activity might play a role in glucose metabolism (Blackman et al., 2013). Collectively, these studies suggest that SLC26A9 is a disease modifier and novel therapeutic target that may compensate for impaired CFTR-mediated Cl^-^ secretion. Recently, dysfunction of Slc26a9-mediated ion composition was associated with Sjögren’s syndrome (SS) onset (Tandon et al., 2017), according to the alteration of the expression and cellular localization of Slc26a9 in SS saliva when compared with healthy controls (Pedersen et al., 2005; Pijpe et al., 2007). Although most studies have focused on the role of Slc26a9 in CF-related disease onset, we anticipate that Slc26a9 may also be involved in the pathogenesis of some unreported diseases, such as duodenal ulcers, Type 2 diabetes and hypertension, based on functional analysis (Amlal et al., 2013; Liu et al., 2015; Li et al., 2016). The role of Slc26a9 remains a mystery in many fields and diseases and merits further and intensive investigation. **Table 1** lists the SNPs in the *slc26a9* gene that are related to different diseases.

**Table 1:** SNPs in the *slc26a9* gene that have been reported to be related to the onset of diseases.

Disease	SNPs in the *slc26a9* gene	References
Schizophrenia	rs11240594	McClay et al., 2011
Asthma	rs2282430	Anagnostopoulou et al., 2012
Meconium ileus	rs4077468 rs7512462	Sun et al., 2012 Li et al., 2014
CF-related diabetes Pancreatic insufficiency and Early exocrine pancreatic disease	rs4077468 rs4077469 rs7512462	Blackman et al., 2013 Meyre and Pare, 2013 Soave et al., 2014 Li et al., 2014 Miller et al., 2015

## Summary and Outlook

Slc26a9 is a member of a relatively new Slc26a gene family with multiple functions. Although extensive work has been performed to investigate the expression pattern, functional diversity, regulatory mechanism and pathophysiology of this novel protein in different organs by various systems, the exact physiological and pathophysiological role of Slc26a9 remains a mystery in many fields. Future work should not only further investigate its regulatory role in ion transport but also elucidate the characteristics of its non-ionic transport, i.e., Slc26a9 is possibly involved in volume control/apoptosis/migration/differentiation. Importantly, previous studies mainly focused on the role of Slc26a9 as a disease modifier of CF-related diseases, based on the overlapping expression pattern and function of the two proteins in some organs. However, an increasing number of functional studies have shown that Slc26a9 may also be involved in some non-CF-related diseases, such as Sjögren’s syndrome, schizophrenia and unreported diseases, including duodenal ulcers, Type 2 diabetes and hypertension. Therefore, basic and genetic research is required in the future to determine whether Slc26a9 can be a clinically relevant disease modifier or promising therapeutic target.

## Author Contributions

XL and TL performed the work and contributed equally. All authors wrote the manuscript.

## Conflict of Interest Statement

The authors declare that the research was conducted in the absence of any commercial or financial relationships that could be construed as a potential conflict of interest.
